# Pneumonia caused by extensive drug-resistant *Acinetobacter baumannii* among hospitalized patients: genetic relationships, risk factors and mortality

**DOI:** 10.1186/s12879-017-2471-0

**Published:** 2017-05-30

**Authors:** Yu jun Li, Chu zhi Pan, Chang quan Fang, Zhu xiang Zhao, Hui ling Chen, Peng hao Guo, Zi wen Zhao

**Affiliations:** 1 The First Affiliated Hospital of Jinan University, the West of Huangpu Street, Guangzhou, China; 2 Department of Respiratory Medicine, Guangzhou First People’s Hospital, Guangzhou Medical University, Panfu Road, Guangzhou, China; 3 Department of Hepatobiliary Surgery, the Third Affiliated Hospital of Sun Yat-sen University, Tian He Road, Guangzhou, China; 4 Department of Respiratory Medicine, Guangzhou Red Cross Hospital, Tong Fu Zhong Road, Guangzhou, China; 5 Department of Clinic Laboratory, Guangzhou First People’s Hospital, Guangzhou Medical University, Panfu Road, Guangzhou, China; 6 Department of Clinic Laboratory, the First Affiliated Hospital of Sun Yat-sen University, Zhong Shan Er Road, Guangzhou, China

**Keywords:** *Acinetobacter baumannii*, Extensive drug resistance, Multilocus sequence typing, *bla*_*OXA-51*-like_ gene, Pneumonia

## Abstract

**Background:**

The clonal spread of multiple drug-resistant *Acinetobacter baumannii* is an emerging problem in China. We analysed the molecular epidemiology of *Acinetobacter baumanni* isolates at three teaching hospitals and investigated the risk factors, clinical features, and outcomes of hospital-acquired pneumonia caused by extensive drug-resistant *Acinetobacter baumannii* (XDRAB) infection in Guangzhou, China.

**Methods:**

Fifty-two *A. baumannii* isolates were collected. Multilocus sequence typing (MLST) was used to assess the genetic relationships among the isolates. The *bla*_*OXA-51-like*_ gene was amplified using polymerase chain reaction (PCR) and sequencing. The resistance phenotypes were determined using the disc diffusion method. A retrospective case-control study was performed to determine factors associated with XDRAB pneumonia.

**Results:**

Most of the 52 *A. baumannii* isolates (*N* = 37, 71.2%) were collected from intensive care units (ICUs). The respiratory system was the most common bodily site from which *A. baumannii* was recovered (*N* = 45, 86.5%). Disc diffusion classified the isolates into 17 multidrug-resistant (MDR) and 35 extensively drug-resistant (XDR) strains. MLST grouped the *A. baumannii* isolates into 5 existing sequence types (STs) and 7 new STs. ST195 and ST208 accounted for 69.2% (36/52) of the isolates. The clonal relationship analysis showed that ST195 and ST208 belonged to clonal complex (CC) 92. According to the sequence-based typing (SBT) of the *bla*_*OXA-51-like*_ gene, 51 *A. baumannii* isolates carried OXA-66 and the rest carried OXA-199. There were no significant differences with respect to the resistance phenotype between the CC92 and non-CC92 strains (*P* = 0.767). The multivariate analysis showed that the APACHE II score, chronic obstructive pulmonary disease (COPD) and cardiac disease were independent risk factors for XDRAB pneumonia (*P* < 0.05). The mortality rate of XDRAB pneumonia was high (up to 42.8%), but pneumonia caused by XDRAB was not associated with in-hospital mortality (*P* = 0.582).

**Conclusions:**

ST195 may be the most common ST in Guangzhou, China, and may serve as a severe epidemic marker. SBT of *bla*_*OXA-51-like*_ gene variants may not result in sufficient dissimilarities to type isolates in a small-scale, geographically restricted study of a single region. XDRAB pneumonia was strongly related to systemic illnesses and the APACHE II score but was not associated with in-hospital mortality.

**Electronic supplementary material:**

The online version of this article (doi:10.1186/s12879-017-2471-0) contains supplementary material, which is available to authorized users.

## Background

*Acinetobacter baumannii* (AB) is one of the most important and common pathogens causing nosocomial outbreaks worldwide, especially in intensive care units (ICUs). The most common bodily site of *A. baumannii* infection is the respiratory tract, particularly in cases of hospital-acquired pneumonia (HAP) [1, 2]. *A. baumannii* is also notorious for its remarkable ability to acquire antibiotic resistance. Data from the CHINET surveillance system demonstrated that *A. baumannii* resistance to many important antimicrobial agents has increased, especially imipenem and meropenem, which increased from 31% in 2005 to 62.4% in 2014 and from 39% in 2005 to 66.7% in 2014, respectively [3]. Recently, the rise in the frequency of nosocomial infections caused by extremely drug resistant (XDR) *A. baumannii* strains (defined as resistance to all available antibiotics except colistin and tigecycline) has been of great concern because XDR resistance has been associated with high mortality and treatment failure [2, 4–7]. According to our previous study [5], most of the isolates (76.2%,32/42) were XDR strains, mostly recovered from the respiratory system, but at present little research concerning extensive drug-resistant *A. baumannii* (XDRAB) pneumonia has been reported.

Currently, *A. baumannii* is recognized as one of the most difficult health care-associated infections to control and treat, and the optimal treatment of infections caused by XDRAB has not been established [6]. Surveillance of *A baumannii* isolates may inform prevention and control measures for these infections. Additionally, determining the process of disease spread by routine surveillance can abrogate routes of bacterial transmission [2]. Multilocus sequence typing (MLST) is a widely used technique for bacterial typing. MLST provides a portable method that is suitable for global epidemiological studies and monitoring of the national and international spread of bacteria [8, 9]. Currently, two large national studies [10, 11] have confirmed that CC92 represents the most epidemic sequence type (ST) in China. ST92, which is the founder of CC92, is the predominant ST, whereas other STs belonging to CC92 vary by area. ST75 may be the most common epidemic ST in eastern China [12], whereas ST138 may be the most common ST in western China [13]. Our previous study discovered that ST195 and ST208 belonged to CC92 were the major clone spreading in our hospital [5]. We assumed that ST195 and ST208 may be more common in southern China Guangzhou area, but this needed to be confirmed further. These differences may be due to the different antibiotic usage habits, which possibly influenced the evolution of ST92. However, little is known about the relationship between antibiotic resistance and certain STs. Although MLST has many advantages, it is a robust scheme that is often time-consuming, expensive, and labour-intensive [14]. Currently, several studies have reported that sequence-based typing (SBT) of *bla*_*OXA-51-like*_ gene variants has potential for application to assess the epidemiological characterization of *A. baumannii* [14–16], but more data are needed.

This study investigated 52 *A. baumannii* isolates from three teaching hospitals in Guangzhou to determine the clonality of the isolates. A case-control study was conducted to evaluate the characteristics, risk factors and outcomes for hospital-acquired XDRAB pneumonia, and the relationship between antibiotic resistance and certain STs was also investigated.

## Methods

### Bacterial isolates and antimicrobial susceptibility testing

From April 2011 to February 2012, A total of 52 *A. baumannii* isolates were collected as part of the standard patient care regimen from three teaching hospitals (Guangzhou First People’s Hospital, Guangzhou Medical University, Panfu Road, Guangzhou, China; the Third Affiliated Hospital of Sun Yat-sen University, Tian He Road, Guangzhou, China; and the First Affiliated Hospital of Sun Yat-sen University, Zhong Shan Er Road, Guangzhou, China). Among the 52 *A. baumannii* isolates, 42 isolates had been reported in our previous study [5]. All *A. baumannii* isolates derived from clinical samples (sputum,bronchoalveolar lavage fluid, blood, cerebrospinal fluid, and urine) were collected from patients hospitalized in the general wards and intensive care units (ICUs), Duplicate isolates from the same patients were excluded. The Vitek 2 (bioMerieux, Inc., Durham, NC, USA) automated microbiology system was used in identification of isolates.

According to Clinical and Laboratory Standards Institute (CLSI; M100-S22, 2012) [17], disc diffusion method were used to detect the susceptibility of 52 *A. baumannii* isolates against 15 antibiotics to determine the resistance phenotype. Isolates that showed resistance or intermediate susceptibility to imipenem, meropenem, amikacin, piperacillin/tazobactam, cefoperazone/sulbactam, ceftazidime, ceftriaxone, cefepime, aztreonam, levofloxacin, ciprofloxacin, doxycycline and tobramycin were considered XDRAB isolates. Melone Pharmaceutical Co. Ltd. (China) provided the antibiotic discs (OXOID). *Escherichia coli* ATCC 25922 and *Pseudomonas aeruginosa* ATCC 27853 were used as the control organisms.

### Molecular epidemiological typing

Multilocus sequence typing (MLST) was performed on *A. baumannii,* according to Bartual et al. [18]. Seven conserved housekeeping genes (gltA, gyrB, gdhB, recA, cpn60, gpi, and rpoD) were amplified and sequencing. The allelic numbers and sequence types (STs) were identified by means of the Pubmlst database [19]. The eBURST algorithm (version 3) [20] was used to assign STs to clonal complexes (CCs) and to assess the genetic relationships among the sequences. Sequence-based typing of the *bla*_*OXA-51-like*_ genes (SBT- *bla*_*OXA-51-like*_ genes) was carried out as follows. The OXA-69A and OXA-69B primers [21], which were external to the *bla*_*OXA-51-like*_ gene, were used to amplify the entire gene sequence, followed by sequencing. The sequences were analysed using BLAST (https://blast.ncbi.nlm.nih.gov/Blast.cgi) to determine the genetic diversity of the *bla*_*OXA-51-like*_ genes [14, 15].

### Case-control study

A retrospective case-control study was performed to evaluate the characteristics, risk factors and outcomes of hospital-acquired XDRAB pneumonia. The cases included patients from whom a XDRAB isolate was isolated from clinical cultures of respiratory secretions and who had been shown to have hospital-acquired pneumonia, including ventilator-associated pneumonia (VAP) defined as pneumonia that occurred more than 48 h after endotracheal intubation [22].

The inclusion criteria consisted of the following: a) diagnosis of pneumonia [23] the presence of new or progressive pulmonary infiltrates in chest radiographs, plus at least two of the following supportive clinical signs: temperature of >38 °C or <35.5 °C, leukocytosis (>12,000 WBC/mm^3^) or leukopenia (<4000 WBC/mm^3^), purulent bronchial secretions, or worsening oxygenation and b) at least two positive respiratory samples for *A. baumannii* : protected specimen brushing cultures 10^3^ cfu/mL, bronchoalveolar lavage (BAL) fluid specimen 10^4^ or 10^5^ cfu/mL, or 10^6^ cfu/mL in an endotracheal aspirate [23]. Patients <18 years of age, patients hospitalization for <48 h and patients with incomplete medical records were excluded from this study, as were patients who had other non- *A. baumannii* -positive cultures in addition to *A. baumannii* to avoid the inclusion of *A. baumannii* colonization.

The controls were randomly selected adult inpatients in the participating hospitals who were diagnosed with non-XDRAB hospital-acquired pneumonia during their hospital stay. The controls were matched to the cases by hospital. Two controls were recruited for each case. Computerized medical, pharmaceutical and microbiological records were reviewed. A specially designed case record form was used to collect demographic and clinical data, including age, gender, underlying diseases, severity of diseases [calculated by the Acute Physiology and Chronic Health Evaluation (APACHE) II score] while admitted to the general wards or ICU, invasive procedures (central venous and/or arterial catheter, urinary catheter, nasogastric tube and mechanical ventilation), duration of stay in the ICU, hospital stay and antibiotic exposure.

### Statistical analysis

Categorical variables were compared using the Chi-square test with Yates correction or Fisher’s exact test. Continuous variables were analysed using the t test. A *P* value <0.05 in a two-tailed test was considered statistically significant. To test the independence of the risk factors for XDRAB pneumonia, significant variables (*P* <0.05) in the univariate analyses were entered into a multivariate logistic regression model. SPSS (version 18.0) was used for all calculations.

## Results

### Characteristics of the 52 *A. baumannii* isolates

Most of the isolates (*N* = 37, 71.2%) were obtained from intensive care units (ICUs). The respiratory system was the most common bodily site from which *A. baumannii* was recovered (*N* = 45, 86.5%), followed by the blood (*N* = 3, 5.8%). Disc diffusion testing (supplementary information) classified the 52 *A. baumannii* isolates into 17 MDR and 35 XDR strains (Table 1).

**Table 1:** Characteristics of the 52 *A. baumannii* isolates

Number of isolates	Hospital	Specimens	Wards	gltA	gyrB	gdhB	recA	cpn60	gpi	rpoD	ST	CC	*bla*_*OXA-51-like*_ genes	Phenotype	XDRAB pneumonia
1	GFPH	Sputum	ICU	1	3	3	2	2	97	3	ST208	92	OXA-66	XDR	Yes
2	GFPH	Blood	ICU	1	3	3	2	2	96	5	STn1	92	OXA-66	XDR	Yes
3	GFPH	Sputum	ICU	1	3	3	2	2	97	3	ST208	92	OXA-66	XDR	Yes
4	GFPH	Blood	ICU	1	3	3	2	2	96	3	ST195	92	OXA-66	XDR	Yes
5	GFPH	BALF	RICU	1	3	3	2	2	96	3	ST195	92	OXA-66	XDR	Yes
6	GFPH	Sputum	RICU	1	3	3	2	2	96	3	ST195	92	OXA-66	MDR	No
7	GFPH	Sputum	Respiratory	1	15	3	2	2	153	3	ST457	92	OXA-66	XDR	No
8	GFPH	Sputum	RICU	1	3	3	2	2	96	3	ST195	92	OXA-66	XDR	Yes
9	GFPH	Sputum	ICU	1	3	3	2	2	96	3	ST195	92	OXA-66	XDR	Yes
10	GFPH	Sputum	Respiratory	1	3	3	2	2	97	3	ST208	92	OXA-66	XDR	yes
11	GFPH	Sputum	Neurosurgery	1	15	3	2	2	96	3	STn2	92	OXA-199	XDR	Yes
12	GFPH	Sputum	RICU	1	3	3	2	2	96	3	ST195	92	OXA-66	MDR	No
13	GFPH	Sputum	Respiratory	1	3	3	2	2	97	3	ST208	92	OXA-66	XDR	No
14	GFPH	Sputum	ICU	1	3	3	2	2	96	3	ST195	92	OXA-66	MDR	No
15	GFPH	BALF	RICU	1	3	3	2	2	96	3	ST195	92	OXA-66	XDR	yes
16	GFPH	Sputum	ICU	1	3	3	2	2	96	3	ST195	92	OXA-66	XDR	yes
17	GFPH	Wound	Gastroenterology	1	3	3	2	2	96	3	ST195	92	OXA-66	XDR	No
18	GFPH	Sputum	Respiratory	1	3	3	2	2	97	3	ST208	92	OXA-66	XDR	No
19	GFPH	Sputum	ICU	1	3	3	2	2	97	3	ST208	92	OXA-66	XDR	yes
20	GFPH	Urine	Geriatrics ICU	21	15	3	2	35	111	4	ST254	singletons	OXA-66	XDR	No
21	GFPH	BALF	RICU	1	3	3	2	2	96	3	ST195	92	OXA-66	XDR	yes
22	GFPH	BALF	RICU	1	3	3	2	2	96	3	ST195	92	OXA-66	MDR	No
23	GFPH	Sputum	Respiratory	1	3	3	2	2	97	3	ST208	92	OXA-66	XDR	yes
24	GFPH	Sputum	ICU	1	3	3	2	2	97	3	ST208	92	OXA-66	MDR	No
25	GFPH	Sputum	RICU	1	3	3	2	2	96	3	ST195	92	OXA-66	XDR	yes
26	GFPH	Sputum	RICU	1	3	3	2	2	96	3	ST195	92	OXA-66	XDR	yes
27	GFPH	Sputum	Neurosurgery	1	81	3	2	2	96	4	STn3	singletons	OXA-66	MDR	No
28	GFPH	Sputum	Respiratory	21	15	3	2	35	111	4	ST254	singletons	OXA-66	XDR	yes
29	GFPH	Sputum	RICU	21	15	3	2	35	111	4	ST254	singletons	OXA-66	XDR	yes
30	GFPH	Sputum	RICU	1	3	3	2	2	96	3	ST195	92	OXA-66	XDR	No
31	GFPH	Sputum	Respiratory	1	3	3	2	2	97	3	ST208	92	OXA-66	XDR	yes
32	GFPH	Sputum	Neurosurgery	1	81	3	2	2	96	4	STn3	singletons	OXA-66	MDR	No
33	GFPH	Sputum	ICU	1	3	3	2	2	96	3	ST195	92	OXA-66	XDR	Yes
34	GFPH	BALF	RICU	1	15	3	2	2	153	3	ST457	92	OXA-66	XDR	Yes
35	GFPH	CSF	ICU	1	3	3	2	2	96	3	ST195	92	OXA-66	XDR	No
36	GFPH	Sputum	Respiratory	21	15	3	2	35	G1	3	STn4	singletons	OXA-66	MDR	No
37	GFPH	Sputum	ICU	1	3	3	2	2	96	3	ST195	92	OXA-66	XDR	No
38	GFPH	Wound	Burn	A1	15	3	2	2	153	4	STn5	92	OXA-66	XDR	No
39	GFPH	Sputum	Geriatrics ICU	21	15	3	2	35	G1	3	STn4	singletons	OXA-66	XDR	No
40	GFPH	Blood	Urinary surgery	1	3	3	2	2	96	3	ST195	92	OXA-66	XDR	No
41	GFPH	Sputum	Nephrology	1	3	3	2	2	97	3	ST208	92	OXA-66	MDR	No
42	GFPH	BALF	RICU	11	65	3	20	37	96	15	STn6	singletons	OXA-66	MDR	No
43	FAH	Sputum	ICU	1	3	3	2	2	96	3	ST195	92	OXA-66	MDR	N/A
44	FAH	Sputum	ICU	1	3	3	2	2	96	3	ST195	92	OXA-66	XDR	N/A
45	FAH	Sputum	ICU	33	31	2	28	1	96	5	STn7	singletons	OXA-66	MDR	N/A
46	FAH	Sputum	ICU	1	3	3	2	2	97	3	ST208	92	OXA-66	MDR	N/A
47	TAH	Sputum	ICU	1	3	3	2	2	96	3	ST195	92	OXA-66	MDR	N/A
48	TAH	Sputum	ICU	1	3	3	2	2	16	3	ST136	92	OXA-66	XDR	N/A
49	TAH	Sputum	ICU	1	3	3	2	2	96	3	ST195	92	OXA-66	MDR	N/A
50	TAH	Sputum	ICU	1	3	3	2	2	97	3	ST208	92	OXA-66	MDR	N/A
51	TAH	Sputum	ICU	1	3	3	2	2	96	3	ST195	92	OXA-66	MDR	N/A
52	TAH	Sputum	ICU	1	3	3	2	2	16	3	ST136	92	OXA-66	XDR	N/A

*GFPH* Guangzhou First People’s Hospital, *FAH* the First Affiliated Hospital of Sun Yat-sen University, *TAH* the Third Affiliated Hospital of Sun Yat-sen University, *BALF* bronchoalveolar lavage fluid, *CSF* cerebrospinal fluid, *ICU* intensive care unit, *RICU* respiratory intensive care unit, *G1* a new allele that has a T → C mutation at nt3 in the gpi111 locus; *A1* a new allele possessing two mutations at the gltA1 locus (A → C mutations at nt156 and nt159); *N/A* not available

### MLST and SBT- *bla*_*OXA-51-like*_ genes

**Fig. 1:**
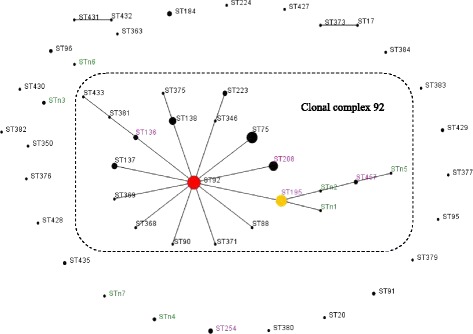
Population snapshot of *A. baumannii* in this study and other existing isolates in China. Population snapshot of *A. baumannii* in this study and existing isolates in China based on the data contained in the Pubmlst database as of 27 April 2013 [5, 18] represented by an eBURST algorithm. Circles represent STs, and their sizes correspond to the numbers of isolates. The *red circle* represents the founder ST (ST92). The broken line indicates clonal complex (CC) 92. The ST labels are coloured as follows: *black*, STs found only in the Pubmlst database; *green*, STs found only in this study; and *purple*, STs found in both the Pubmlst database and this study. ST254, STn3, STn4, STn6 and STn7 were the singletons in this study

According to the MLST, 52 *A. baumannii* isolates were grouped into 12 distinct STs, including 5 existing STs and 7 novel STs (STn1 to STn7). STn4 carried allele G1 with a T → C mutation at the 3rd nucleotide site (nt3) on the gpi111 locus. STn5 carried allele A1 with an A → C mutation at nt156 and nt159 on the gltA1 locus. ST195 and ST208 were the most common STs, accounting for 69.2% of all isolates. The clonal relationship analysis showed that ST195 and ST208 belonged to CC92. According to the sequence-based typing of the *bla*_*OXA-51-like*_ genes, 51 *A. baumannii* isolates carried OXA-66 and the rest carried OXA-199. No significant differences with respect to the resistance phenotype were detected between the CC92 and non-CC92 strains (*P* = 0.767) (Tables 1 and 2 and Fig. 1).

**Table 2:** The relationship between the clonal complex (CC) and resistance phenotype of the 52 *A. baumannii* isolates

Clonal complex	No. isolates	MDR N (%)	XDR N (%)	Statistical analysis^a^	
				χ2 -values	*P* values
CC92	43	14 (32.6)	29 (67.4)	0.088	0.767
ST195	24	9	15		
ST208	12	5	7		
ST457	2	0	2		
ST136	2	0	2		
STn1	1	0	1		
STn2	1	0	1		
STn5	1	0	1		
Non-CC92	9	4 (44.4)	5 (55.6)		
ST254	3	0	3		
STn3	2	2	0		
STn4	2	0	2		
STn6	1	1	0		
STn7	1	1	0		

^a^ comparison of CC92 with non-CC92 strains

### Case-control study

Full medical records were available for 42 of the 52 patients from whom the *A. baumannii* isolates were isolated. A total of 32 patients were diagnosed with XDRAB acquisition, and 21 patients finally met the inclusion criteria and were assessed in the case-control study. Of the 21 patients with XDRAB pneumonia, 17 were men and four were women. The mean age was 77.5 years (standard deviation 11.6 years). Sixteen patients were receiving care in ICUs and 5 were in general wards during specimen collection (Tables 1 and 3).

**Table 3:** Comparison of clinical data for pneumonia-related characteristics in HAP patients with XDRAB and non-XDRAB

	XDRAB (*N* = 21) *N* (%)	Non-XDRAB (*N* = 42) *N* (%)	*P*-value
Age, y^a^	77.5 ± 11.6	68.6 ± 18.4	0.023
Gender (M/F), n	17/4	23/19	0.079
APACHE II score^a^	21.9 ± 6.8	18.0 ± 4.9	0.011
Related to hospitalization^a^	18 (85.7)	30 (71.4)	0.347
Days of mechanical ventilation before XDRAB (days)	10.5 ± 11.6	5.2 ± 5.8	0.059
Hospital days before XDRAB (days)	18.3 ± 11.3	12.6 ± 11.2	0.064
Length of stay in the ICU (days)	30.1 ± 20.0	21.4 ± 21.7	0.127
Length of stay in the hospital (days)	45.5 ± 28.8	38.5 ± 24.2	0.199
Associated disease, n (%)			
COPD	13 (61.9)	9 (21.4)	0.001
Diabetes mellitus	2 (9.5)	10 (40.4)	0.307
Malignancy	2 (9.5)	9 (21.4)	0.411
Cardiac disease	13 (61.9)	6 (14.2)	0.000
Renal disease	5 (23.8)	2 (4.7)	0.065
Neurological disease	8 (38.0)	22 (52.3)	0.422
Device, n (%)			
Urinary catheter	21 (100.0)	36 (85.7)	0.172
Nasogastric tube	21 (100.0)	36 (85.7)	0.172
Mechanical ventilation	16 (76.1)	31 (73.8)	1.000
Drug usage, n (%)			
Glucocorticoids	10 (47.6)	18 (42.8)	0.720
PPIs	14 (66.7)	32 (76.1)	0.422
Antimicrobial n (%)			
Cephalosporin			
Second generation	3 (14.2)	5 (11.9)	1.000
Third generation	7 (33.3)	18 (42.8)	0.649
β-lactamase inhibitor	20 (95.2)	31 (73.8)	0.089
Quinolone	13 (61.9)	19 (45.2)	0.327
Aminoglycoside	2 (9.5)	4 (9.5)	1.000
Carbapenem	11 (52.3)	11 (26.1)	0.040
Antimicrobial Combination therapy, n (%)	11 (52.3)	19 (45.2)	0.593
Mortality, n (%)	9 (42.8)	15 (35.7)	0.582

^a^Values are presented as the mean ± standard deviation; malignancy includes haematological malignancies and solid tumours; cardiac disease includes coronary artery disease, hypertensive heart disease, valvular disease and cardiomyopathy; renal disease includes chronic renal failure; neurological disease includes cerebral haemorrhage and cerebral infarction; *PPIs* proton pump inhibitor drugs

The potential risk factors for patients with XDRAB pneumonia are shown in Table 3. The two groups were similar with respect to gender, days of mechanical ventilation before XDRAB pneumonia, days spent in the hospital before XDRAB pneumonia, length of stay in the ICU, and length of stay in the hospital. Additionally, no significant differences were detected with respect to malignancy, renal disease, neurological disease, urinary catheter, nasogastric tube, mechanical ventilation, or the use of glucocorticoids, PPIs, cephalosporin, β-lactamase inhibitor, quinolone or minoglycoside. The mortality rate of XDRAB pneumonia was high (up to 42.8%), but pneumonia caused by XDRAB was not associated with in-hospital mortality (*P* = 0.582). Compared with the non-XDRAB patients, the patients with XDRAB pneumonia were significantly more likely to be older (77.5 ± 11.6 vs 68.6 ± 18.4 years, *P* = 0.023) and have a higher initial severity of illness at admission as indicated by the higher APACHE II score (21.9 ± 6.8 vs 18.0 ± 4.9, *P* = 0.011). Moreover, chronic obstructive pulmonary disease (COPD), cardiac disease and carbapenem use were risk factors for XDRAB pneumonia (*P* < 0.05).

The multivariate analysis using a logistic regression model results are presented in Table 4. The APACHE II score (OR, 1.17; 95% CI: 1.01–1.35, *P* = 0.034), COPD (OR, 7.25; 95% CI: 1.54–33.9, *P* = 0.012), and cardiac disease (OR, 6.94; 95% CI: 1.43–33.6, *P* = 0.016) were identified as independent risk factors for XDRAB acquisition.

**Table 4:** Multi-variate analysis of risk factors for patients with XDRAB pneumonia

Risk factor	OR (95% CI)	*P*-value
APACHE II score	1.17 (1.01–1.35)	0.034
COPD	7.25 (1.54–33.9)	0.012
Cardiac disease	6.94 (1.43–33.6)	0.016

## Discussion

In this study, we examined the molecular typing and characteristics of *A. baumannii* at three teaching hospitals. In the MLST, CC92 was the most prevalent clonal complex. However, ST92, which was the predicted founder of CC92 and was reported to be one of the most epidemic STs in multiple provinces in China [1, 10, 11], was not detected in our study. ST92 was also not detected in other studies from China [1, 5] and South Africa [24]. The lack of ST92 detection may be due to the relatively small sample size in our study, which may not accurately represent the diversity and relative abundance of *A. baumannii* STs. However, some studies [1, 5, 25] have shown that ST195, ST208, ST365, and ST191 (but not ST92) are the most common STs discovered in different hospitals across China. In this study, ST195 was the most commonly observed ST, accounting for 24/52 (46.1%) of the isolates, follow by ST208. These findings are similar to the findings of Zhou et al. [25], who found that ST195 accounted for 31/57 (54.4%) isolates. These data collectively suggest that ST195 is the most common ST and may serve as a severe epidemic marker in the Guangzhou area.

Some data are available to clarify why CC92 is predominant. Zhong et al. [12] found that ST75, which is the single locus variant (SLV) of ST92, differs in its gpi loci and belongs to CC92, has more severe imipenem resistance that might improve its chances of survival. In our study, CC92 accounted for 82.7% (43/52) of the isolates. Most of the XDR isolates (29/34, 85.3%) were from CC92. However, CC92 included 14 MDR isolates with less severe resistance, and non-CC92 had similar results. No significant differences with respect to the resistance phenotype were detected between the CC92 and non-CC92 strains (*P* = 0.767). Runnegar et al. [26] revealed that CC92 had been spread in the hospital for 9 years with variable antibiotic susceptibility. These findings may suggest that both adaptation to the hospital environment and antibiotic resistance have been important for the success of CC92.

The *bla*_*OXA-51-like*_ genes are unique to *A. baumannii* and immobile and thus may be used as markers for the identification of this species [21]. Recently, sequence-based typing (SBT) of *bla*_*OXA-51-like*_ gene variants was reported to have potential for use in the epidemiological characterization of *A. baumannii* isolates obtained from various locations in Europe. *bla*_*OXA-69*_, *bla*_*OXA-66*_, and *bla*_*OXA-71*_ are the predominant members of closely related *bla*_*OXA-51-like*_ subgroups, which are associated with European clone I (EUI), EUII, and EUIII, respectively [14–16]. However, few studies have investigated the correlation between *bla*_*OXA-51-like*_ variants with MLST typing.

According to a study by Hamouda et al. [14], MLST data showed that all isolates harbouring the major *bla*_*OXA-51-like*_ alleles (OXA-66, OXA-69, and OXA-71) fell within the three major European clonal lineages. The SBT-*bla*_*OXA-51-like*_ gene scheme produced results comparable to those produced by the Bartual MLST for the identification of the major epidemic lineages. Pournaras et al. [27] evaluated the SBT-*bla*_*OXA-51-like*_ gene scheme in parallel with Pasteur’s MLST. In the study, according to the SBT-*bla*_*OXA-51-like*_ gene, all 585 *A. baumannii* isolates from a large international collection were typed and assigned correctly to the nine CCs and the singleton ST78. Zhou et al. [25] revealed that 52 isolates from Guangzhou city in China carrying bla_*OXA-66*_ were assigned to six distinct STs, which clustered into CC92; the remaining isolates belonged to four singletons that each carried a single *bla*_*OXA-51-like*_ allele. The results of these studies indicate that the SBT-*bla*_*OXA-51-like*_ gene scheme has the advantage of a significantly reduced sequencing cost and assay time and may be effective for the rapid typing of *A. baumannii* strains. However, in our study, only one isolate carried OXA-199, whereas the remaining 51 *A. baumannii* isolates carried OXA-66. The SBT-*bla*_*OXA-51-like*_ gene scheme failed to discriminate strains carrying the same OXA-66 allele. Similar findings reported by Wang et al. [28] showed that 18 representative isolates from different hospitals across China carried the same OXA-66 allele.

In Hamouda et al.’s study [14], the SBT-*bla*_*OXA-51-like*_ method was evaluated in a large international collection of *A. baumannii* isolates including 22 countries. Similarly, Pournaras et al. [20] typed isolates obtained from various locations in Europe (Italy, Greece, Turkey, and Lebanon). Environmental differences among countries may act as natural selection forces [12] to introduce diversity among *bla*_*OXA-51-like*_ genes. When confined to one city or hospital, the controlled environment and antibiotic usage habits might influence the convergent evolution of the *bla*_*OXA-51-like*_ genes. As a result, single-locus sequence-based typing of the *bla*_*OXA-51-like*_ genes may not be effective at distinguishing isolates in a small-scale, geographically restricted study. However, further investigation is needed to confirm this hypothesis.

Some studies have reported risk factors for antibiotic resistance in *A. baumannii* infection, which include the length of stay in an ICU, severity of the underlying disease, mechanical ventilation, invasive procedures, and prior antibiotic use [29–32]. However, the clinical characteristics of XDRAB pneumonia have rarely been reported. In our study, univariate analysis was used to identify patients at risk of acquiring XDRAB pneumonia and showed that patients with XDRAB were older and had higher APACHE II scores than patients without XDRAB, which was similar to the findings of Özgür et al. [33]. Additionally, patients who had COPD and cardiac diseases were more likely to acquire XDRAB. Multivariate analysis using a logistic regression model showed that the APACHE II score, COPD and cardiac disease were independent risk factors for XDRAB pneumonia development. Thus, XDRAB may be particularly pathogenic in patients who are immunocompromized. Our study found that carbapenem use was an important risk factor for XDRAB pneumonia (*P* = 0.04). Previous studies [29, 31, 34] have also shown that selective pressure exerted by the use of carbapenem leads to the emergence of multidrug resistant (MDR) and XDR *A. baumannii* isolates. Therefore, rational use of carbapenem is necessary to reduce the risk of generating resistant mutants.

High mortality rates have been reported for nosocomial pneumonia caused by *A. baumannii* (ranging from 28.1 to 85.3%). The independent risk factors included the severity of illness (e.g., severe sepsis, septic shock and APACHE II score) and empiric inappropriate therapy [31, 33, 35, 36]. In one study, patients with carbapenem-resistant *A. baumannii* (CRAB) pneumonia had a higher mortality rate than patients with carbapenem-susceptible *A. baumannii* (CSAB) pneumonia based on the survival analysis (29.9% vs 45.6%, respectively, *P* = 0.02) [31]. However, another study reported mortality rates for patients with MDR *A. baumannii* infections that were not significantly higher than the mortality rates in patients without MDR *A. baumannii* infections [37]. Furthermore, in a study on ventilator-associated pneumonia (VAP) due to XDRAB, the mortality rates were not significantly higher than those of non-XDRAB VAP in ICU patients [33]. Our study showed a high hospital mortality rate in patients with XDRAB pneumonia, but *A. baumannii* resistance was not associated with mortality (*P* = 0.582).

## Conclusions

ST195 may be the most common ST in the Guangzhou region of China and may serve as a severe epidemic marker in this region. SBT of *bla*_*OXA-51-like*_ gene variants may not be able to sufficiently distinguish isolates obtained from a small-scale, geographically restricted study. XDRAB pneumonia was strongly related to systemic illnesses and the APACHE II score but was not associated with in-hospital mortality.

## Additional file

Additional file 1: 52 Isolates were tested against antibiotics by disc diffusion. All 52 of the isolates were tested against a panel of antibiotics with the disc diffusion method, as recommended by the Clinical and Laboratory Standards Institute (CLSI;M100-S22, 2012) [17], to determine the resistance phenotype. Multidrug resistance (MDR) was defined as acquired non-susceptibility to at least one agent in three or more antimicrobial categories, and extensive drug resistance (XDR) was defined as resistance to all available antibiotics except colistin and tigecycline [5]. GFPH, Guangzhou First People’s Hospital; FAH, the First Affiliated Hospital of Sun Yat-sen University; TAH, the Third Affiliated Hospital of Sun Yat-sen University; R, resistance; S, sensitivity; I, intermediate; PB, polymyxin B (300 units); TGC, tigecycline (15 μg); IPM, imipenem (10 μg); AK, amikacin (30 μg); TOB, tobramycin (10 μg); MEM, meropenem (10 μg); TZP, piperacillin/tazobactam (100∕10 μg); SCF, cefoperazone/sulbactam (70∕35 μg); CAZ, ceftazidime (30 μg); CRO, ceftriaxone (30 μg); FEP, cefepime (30 μg); ATM, aztreonam (30 μg); LEV, levofloxacin (5 μg); CIP, ciprofloxacin (5 μg); DO, doxycycline (30 μg). (DOCX 24 kb)

## Abbreviations

AB: *Acinetobacter baumannii*
APACHE II score: Acute Physiology and Chronic Health Evaluation II score
ATS/IDSA: American Thoracic Society/Infectious Diseases Society of America
BAL: Bronchoalveolar lavage
CC: Clonal complex
CLSI: Clinical and Laboratory Standards Institute
COPD: Chronic obstructive pulmonary disease
CRAB: Carbapenem-resistant *A. baumannii*
CSAB: Carbapenem-susceptible *A. baumanni*i
ICUs: Intensive care units
MDR: Multidrug resistant
MLST: Multilocus sequence typing
PCR: Polymerase chain reaction
SBT: Sequence-based typing
ST: Sequence type
VAP: Ventilator-associated pneumonia
XDRAB: Extensive drug-resistant *Acinetobacter baumannii*
