# Supplementary material for: Pneumonia caused by extensive drug-resistant Acinetobacter baumannii among hospitalized patients: genetic relationships, risk factors and mortality
Source: BMC Infect Dis. 2017 May 30;17:371. doi: 10.1186/s12879-017-2471-0 (PMC5450129; doi:10.1186/s12879-017-2471-0)
Supplement: Additional file 1: — 52 Isolates were tested against antibiotics by disc diffusion. All 52 of the isolates were tested against a panel of antibiotics with the disc diffusion method, as recommended by the Clinical and Laboratory Standards Institute (CLSI;M100-S22, 2012) [17], to determine the resistance phenotype. Multidrug resistance (MDR) was defined as acquired non-susceptibility to at least one agent in three or more antimicrobial categories, and extensive drug resistance (XDR) was defined as resistance to all available antibiotics except colistin and tigecycline [5]. GFPH, Guangzhou First People’s Hospital; FAH, the First Affiliated Hospital of Sun Yat-sen University; TAH, the Third Affiliated Hospital of Sun Yat-sen University; R, resistance; S, sensitivity; I, intermediate; PB, polymyxin B (300 units); TGC, tigecycline (15 μg); IPM, imipenem (10 μg); AK, amikacin (30 μg); TOB, tobramycin (10 μg); MEM, meropenem (10 μg); TZP, piperacillin/tazobactam (100∕10 μg); SCF, cefoperazone/sulbactam (70∕35 μg); CAZ, ceftazidime (30 μg); CRO, ceftriaxone (30 μg); FEP, cefepime (30 μg); ATM, aztreonam (30 μg); LEV, levofloxacin (5 μg); CIP, ciprofloxacin (5 μg); DO, doxycycline (30 μg). (DOCX 24 kb) [file 12879_2017_2471_MOESM1_ESM.docx]

**Additional file 1: 52 Isolates were tested against antibiotics by disc diffusion***

| Isolate No. | hospital | SCF | TZP | CAZ | CRO | FEP | ATM | IPM | MEM | LEV | CIP | AK | DO | TGC | PB | TOB | Phenotype |
| --- | --- | --- | --- | --- | --- | --- | --- | --- | --- | --- | --- | --- | --- | --- | --- | --- | --- |
| 1 | GFPH | R | R | R | R | R | R | R | R | R | R | R | R | S | S | R | XDR |
| 2 | GFPH | R | R | R | R | R | R | R | R | R | R | R | R | S | S | R | XDR |
| 3 | GFPH | R | R | R | R | R | R | R | R | R | R | R | R | S | S | R | XDR |
| 4 | GFPH | R | R | R | R | R | R | R | R | R | R | R | R | I | S | R | XDR |
| 5 | GFPH | R | R | R | R | R | R | R | R | R | R | R | R | I | S | R | XDR |
| 6 | GFPH | R | R | R | R | R | R | R | R | R | R | S | R | I | S | S | MDR |
| 7 | GFPH | R | R | R | R | R | R | R | R | R | R | R | R | I | S | R | XDR |
| 8 | GFPH | R | R | R | R | R | R | R | R | R | R | R | R | I | S | R | XDR |
| 9 | GFPH | R | R | R | R | R | R | R | R | R | R | R | R | I | S | R | XDR |
| 10 | GFPH | R | R | R | R | R | R | R | R | R | R | R | R | I | S | R | XDR |
| 11 | GFPH | R | R | R | R | R | R | R | R | R | R | R | R | I | S | R | XDR |
| 12 | GFPH | R | R | R | R | R | R | R | R | R | R | S | R | I | S | S | MDR |
| 13 | GFPH | R | R | R | R | R | R | R | R | R | R | R | R | I | S | R | XDR |
| 14 | GFPH | S | R | R | R | R | R | I | I | R | R | R | R | S | S | R | MDR |
| 15 | GFPH | R | R | R | R | R | R | R | R | R | R | R | R | I | S | R | XDR |
| 16 | GFPH | R | R | R | R | R | R | R | R | R | R | R | R | R | S | R | XDR |
| 17 | GFPH | R | R | R | R | R | R | R | R | R | R | R | R | I | S | R | XDR |
| 18 | GFPH | R | R | R | R | R | R | R | R | R | R | R | R | I | S | R | XDR |
| 19 | GFPH | R | R | R | R | R | R | R | R | R | R | R | R | I | S | R | XDR |
| 20 | GFPH | I | R | R | R | R | R | R | R | R | R | R | I | S | S | R | XDR |
| 21 | GFPH | R | R | R | R | R | R | R | R | R | R | R | R | R | S | R | XDR |
| 22 | GFPH | R | R | R | R | R | R | R | R | R | R | S | R | R | S | S | MDR |
| 23 | GFPH | I | R | R | R | R | R | R | R | R | R | R | R | S | S | R | XDR |
| 24 | GFPH | S | R | R | R | R | R | S | S | R | R | S | R | I | S | S | MDR |
| 25 | GFPH | R | R | R | R | R | R | R | R | R | R | R | R | S | S | R | XDR |
| 26 | GFPH | R | R | R | R | R | R | R | R | R | R | R | R | S | S | R | XDR |
| 27 | GFPH | R | R | R | R | R | R | S | I | R | R | R | R | I | S | R | MDR |
| 28 | GFPH | I | R | R | R | R | R | R | R | R | R | R | R | S | S | R | XDR |
| 29 | GFPH | I | R | R | R | R | R | R | R | R | R | R | I | S | S | R | XDR |
| 30 | GFPH | R | R | R | R | R | R | R | R | R | R | R | R | I | S | R | XDR |
| 31 | GFPH | R | R | R | R | R | R | R | R | R | R | R | R | I | S | R | XDR |
| 32 | GFPH | I | R | R | R | I | R | S | S | R | R | R | I | I | S | R | MDR |
| 33 | GFPH | R | R | R | R | R | R | R | R | R | R | R | R | I | S | R | XDR |
| 34 | GFPH | R | R | R | R | R | R | R | R | R | R | R | R | I | S | R | XDR |
| 35 | GFPH | R | R | R | R | R | R | R | R | R | R | R | R | I | S | R | XDR |
| 36 | GFPH | I | R | R | R | R | I | R | R | R | R | R | S | S | S | R | MDR |
| 37 | GFPH | R | R | R | R | R | R | R | R | R | R | R | R | I | S | R | XDR |
| 38 | GFPH | R | R | R | R | R | R | R | R | R | R | R | R | I | S | R | XDR |
| 39 | GFPH | R | R | R | R | R | R | R | R | R | R | R | I | S | S | R | XDR |
| 40 | GFPH | R | R | R | R | R | R | R | R | R | R | R | R | I | S | R | XDR |
| 41 | GFPH | I | R | R | R | R | R | S | I | R | R | R | R | R | S | R | MDR |
| 42 | GFPH | I | S | R | R | R | R | S | S | I | R | R | I | R | S | R | MDR |
| 43 | FAH | I | R | R | R | I | R | S | S | R | R | R | I | S | S | R | MDR |
| 44 | FAH | R | R | R | R | R | R | R | R | R | R | R | R | I | S | R | XDR |
| 45 | FAH | S | R | R | R | R | R | S | S | R | R | S | R | S | S | S | MDR |
| 46 | FAH | S | R | R | R | R | R | S | S | R | R | R | R | S | S | R | MDR |
| 47 | TAH | S | R | R | R | R | R | I | I | R | R | S | R | S | S | S | MDR |
| 48 | TAH | R | R | R | R | R | R | R | R | R | R | R | R | S | S | R | XDR |
| 49 | TAH | R | R | R | R | R | R | S | I | R | R | R | R | S | S | R | MDR |
| 50 | TAH | R | R | R | R | R | R | S | I | R | R | R | R | S | S | R | MDR |
| 51 | TAH | I | R | R | R | R | I | R | R | R | R | R | S | S | S | R | MDR |
| 52 | TAH | R | R | R | R | R | R | R | R | R | R | R | R | I | S | R | XDR |

*All 52 of the isolates were tested against a panel of antibiotics with the disc diffusion method, as recommended by the Clinical and Laboratory Standards Institute (CLSI;M100-S22, 2012) [17], to determine the resistance phenotype. Multidrug resistance (MDR) was defined as acquired non-susceptibility to at least one agent in three or more antimicrobial categories, and extensive drug resistance (XDR) was defined as resistance to all available antibiotics except colistin and tigecycline[5]. GFPH, Guangzhou First People’s Hospital; FAH, the First Affiliated Hospital of Sun Yat-sen University; TAH, the Third Affiliated Hospital of Sun Yat-sen University; R, resistance; S, sensitivity; I, intermediate; PB, polymyxin B (300 units); TGC, tigecycline (15 μg); IPM, imipenem (10 μg); AK, amikacin (30 μg); TOB, tobramycin (10 μg); MEM, meropenem (10 μg); TZP, piperacillin/tazobactam (100∕10 μg); SCF, cefoperazone/sulbactam (70∕35 μg); CAZ, ceftazidime (30 μg); CRO, ceftriaxone (30 μg); FEP, cefepime (30 μg); ATM, aztreonam (30 μg); LEV, levofloxacin (5 μg); CIP, ciprofloxacin (5 μg); DO, doxycycline (30 μg).
